# A very simple, re-executable neuroimaging publication

**DOI:** 10.12688/f1000research.10783.2

**Published:** 2017-06-15

**Authors:** Satrajit S. Ghosh, Jean-Baptiste Poline, David B. Keator, Yaroslav O. Halchenko, Adam G. Thomas, Daniel A. Kessler, David N. Kennedy

**Affiliations:** 1 McGovern Institute for Brain Research, Massachusetts Institute of Technology: MIT, Cambridge, MA, USA; 2 Department of Otology and Laryngology, Harvard Medical School, Boston, MA, USA; 3 Henry Wheeler Jr. Brain Imaging Center, Helen Wills Neuroscience Institute, University of California, Berkeley, Berkeley, CA, USA; 4 Department of Psychiatry and Human Behavior, Department of Computer Science, Department of Neurology, University of California, Irvine, Irvine, CA, USA; 5 Department of Psychological and Brain Sciences, Dartmouth College, Hanover, NH, USA; 6 Data Science and Sharing Team, National Institute of Mental Health, Intramural Research Programs, Bethesda, MD, USA; 7 Department of Psychiatry and Department of Radiology, University of Michigan, Ann Arbor, MI, USA; 8 Eunice K. Shriver Center and Department of Psychiatry, University of Massachusetts Medical School, Worcester, MA, USA

**Keywords:** Neuroimaging analysis, re-executable publication, reproducibility

## Abstract

Reproducible research is a key element of the scientific process. Re-executability of neuroimaging workflows that lead to the conclusions arrived at in the literature has not yet been sufficiently addressed and adopted by the neuroimaging community. In this paper, we document a set of procedures, which include supplemental additions to a manuscript, that unambiguously define the data, workflow, execution environment and results of a neuroimaging analysis, in order to generate a verifiable re-executable publication. Re-executability provides a starting point for examination of the generalizability and reproducibility of a given finding.

## Introduction

The quest for more reproducibility and replicability in neuroscience research spans many types of problems. True reproducibility requires the observation of a ‘similar result’ through the execution of a subsequent independent, yet similar, analysis on similar data. However, what constitutes ‘similar’, and how to appropriately annotate and integrate a lack of replication in specific studies remains a problem for the community and the literature that we generate.

### The reproducibility problem

A number of studies have brought the reproducibility of science into question ( Prinz *et al.*, 2011). Numerous factors are critical to understand reproducibility, including: sample size, and its related issues of power and generalizability ( Button *et al.*, 2013; Ioannidis, 2005); P-hacking, trying various statistical approaches in order to find analyses that reach significance ( Simmons *et al.*, 2011; Simonsohn *et al.*, 2014); completeness of methods description, the written text of a publication cannot completely describe an analytic method in its entirety. Coupled with this is the publication bias that arises from only publishing results from the positive (“significant”) tail of the distribution of findings. This contributes to a growing literature of findings that do not properly ‘self-correct’ through an equivalent publication of negative findings (that indicate a lack of replication). Such corrective aggregation is needed to balance the inevitable false positives that result from the millions of experiments that are performed each year.

But before even digging too deeply into the exceedingly complex topic of reproducibility, there already is great concern that a typical neuroimaging publication, the basic building block that our scientific knowledge enterprise is built upon, is rarely even re-executable, even by the original investigators. The general framework for a publication is the following: take some specified “Data”, apply a specified “Analysis”, and generate a set of “Results”. From the Results, claims are then made and discussed. In the context of this paper, we consider “Analysis” to include the software, workflow and execution environment, and use the following definitions of reproducibility:

- **Re-executability (publication-level replication):** The exact same data, operated on by the exact same analysis should yield the exact same result. This is currently a problem since publications, in order to maintain readability, do not typically provide a complete specification of the analysis method or access to the exact data.
- **Generalizability:** We can divide generalizability into three variations:
  - ***Generalization Variation 1:*** Exact Same Data + ***Nominally ‘Similar’ Analyses*** should yield a ‘Similar’ Result (i.e. FreeSurfer subcortical volumes compared to FSL FIRST)
  - ***Generalization Variation 2: Nominally ‘Similar’ Data*** + Exact Same Analysis should yield a ‘Similar’ Result (i.e. the cohort of kids with autism I am using compared to the cohort you are using)
  - ***Generalized Reproducibility:*** Nominally ‘Similar’ Data + Nominally ‘Similar’ Analyses should yield a ‘Similar’ Result

Since we do not really characterize data, analysis, and results very exhaustively in the current literature, this lack of provenance ( Mackenzie-Graham *et al.*, 2008) permits the concept of ‘similar’ to have lots of wiggle room for interpretation (both to enhance similarity and to discount differences, as desired by the interests of the author).

In this paper, we look more closely at the re-executability necessary for publication-level replication. The technology exists, in many cases, to make neuroimaging publications that are fully re-executable. Re-executability of an initial publication is a crucial step in the goal of overall reproducibility of a given research finding. There are already examples of re-executable individual articles (e.g. Waskom *et al.*, 2014), as well as journals that propose to publish reproducible and open research (e.g. https://rescience.github.io). Here, we propose a formal template for a reproducible brain imaging publication and provide an example on fully open data from the NITRC Image Repository. The key elements to publication re-executability are definition of and access to: 1) the data; 2) the processing workflow; 3) the execution environment; and 4) the complete results. In this report, we use existing technologies (i.e., NITRC ( http://nitrc.org), NIDM ( http://nidm.nidash.org), Nipype ( http://nipy.org/nipype), NeuroDebian ( http://neuro.debian.net)) to generate a re-executable publication for a very simple analysis problem, which can form an essential template to guide future progress in enhancing re-executability of workflows in neuroimaging publications. Specifically, we explore the issue of exact re-execution (identical execution environment) and re-execution of identical workflow and data in ‘similar’ execution environments ( Glatard *et al.*, 2015; Gronenschild *et al.*, 2012).

## Methods

### Overview

We envision a ‘publication’ with four supplementary files, the: 1) data file, 2) workflow file, 3) execution environment specification, and 4) results. The task the author would like to enable, for an interested reader, will be to facilitate the use of the first three specifications and easily be able to run them, and confirm (or deny) the similarity of the results from an independent re-execution compared to those published.

For the purpose of this report, we wanted an easy to execute query run on completely open, publically available data. We also wanted to use a relatively simple workflow that could be run in a standard computational environment and have it operate on a tractable number of subjects. We selected a workflow and sample size such that the overall processing could be accomplished in a few hours. The complete workflow and results can be found in the Github repository (doi, 10.5281/zenodo.800758; Ghosh *et al.*, 2017).

**Figure 1.**
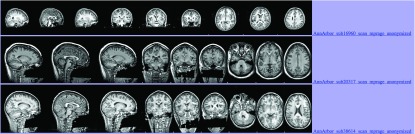
Example images from a subset of three of the subject image datasets used.

***The data.*** The dataset for this exercise was created by a query as an unregistered guest user of the NITRC Image Repository (NITRC-IR; RRID:SCR_004162; Kennedy *et al.*, 2016). We queried the NITRC-IR search page ( http://www.nitrc.org/ir/app/template/Index.vm; 1-Jan-2017) on the ‘MR’ tab with the following specification: age, 10–15 years old; Field Strength, 3. This query returned 24 subjects, which included subject identifier, age, handedness, gender, acquisition site, and field strength. We then selected the ‘mprage_anonymized’ scan type and ‘NIfTI’ file format in order to access the URLs (uniform resource locators) for the T1-weighted structural image data of these 24 subjects. The subjects had the following characteristics: age=13.5 +/- 1.4 years; 16 males, 8 females; 8 right handed, 1 left and 15 unknown. All of these datasets were from the 1000 Functional Connectomes project ( Biswal *et al.*, 2010), and included 9 subjects from the Ann Arbor sub-cohort, and 15 from the New York sub-cohort. We captured this data in tabular form ( Supplementary File 1). Following the recommendations of the Joint Declaration of Data Citation Principles ( Starr *et al.*, 2015), we used the Image Attribution Framework ( Honor *et al.*, 2016) to create a unique identifier for this data collection (image collection: doi, 10.18116/C6C592; Kennedy, 2017). Data collection identifiers are useful in order to track and attribute future reuse of the dataset and maintain the credit and attribution connection to the constituent images of the collection which may, in general, come from heterogeneous sources. Representative images from this collection are shown in Figure 1.

***The workflow.*** For this example, we use a simple workflow designed to generate subcortical structural volumes. We used the following tools from the FMRIB software library, version 5.0.9 (FSL, RRID:SCR_002823; Jenkinson *et al.*, 2012), conformation of the data to FSL standard space (fslreorient2std), brain extraction (BET), tissue classification (FAST), and subcortical segmentation (FIRST).

**Figure 2.**
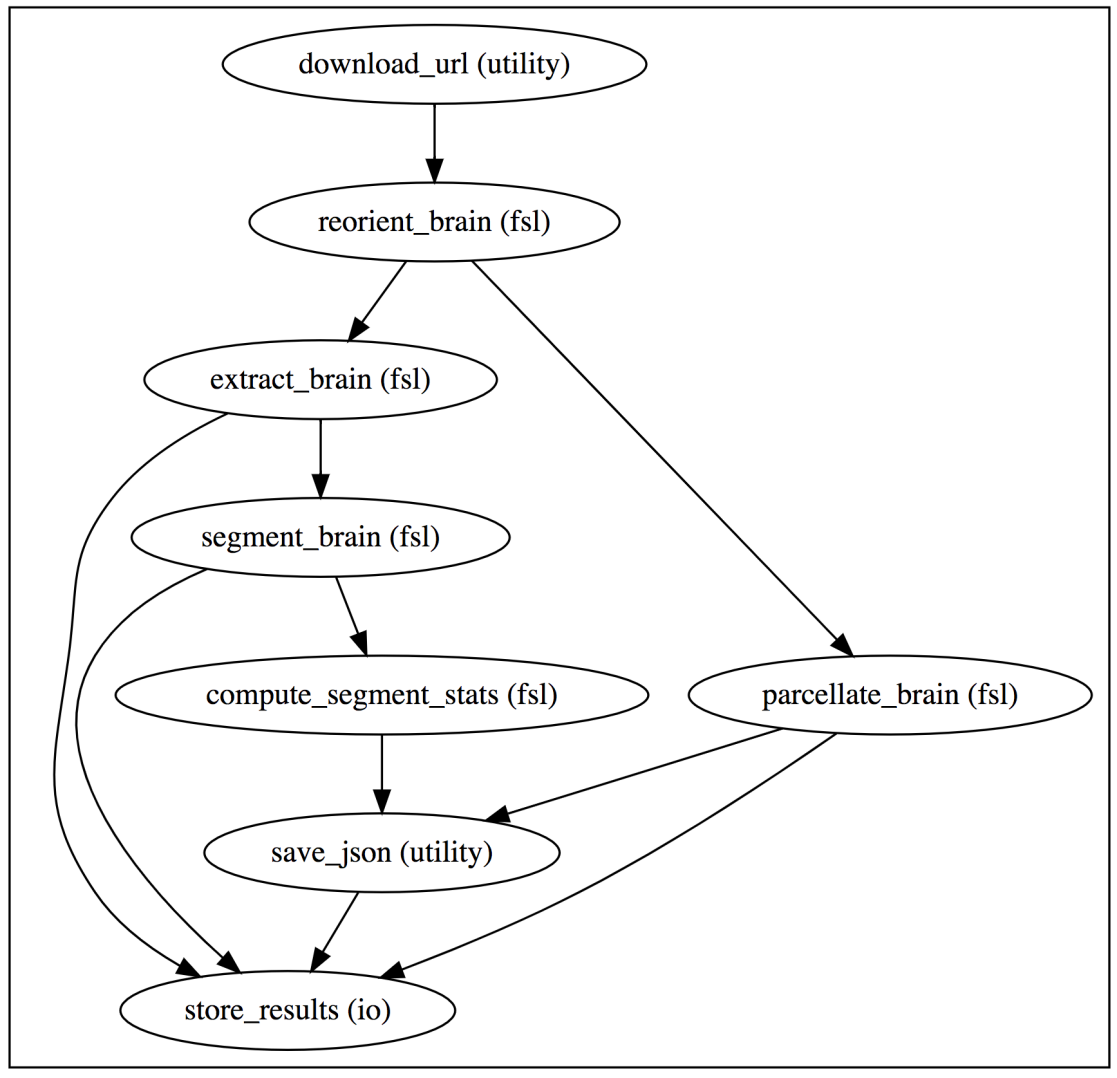
Workflow diagram. The sequence and dependence of processing events used in this example re-executable publication.

This workflow is represented in Nipype (RRID:SCR_002502; Gorgolewski *et al.*, 2011) to facilitate workflow execution and provenance tracking. The workflow is available in the GitHub repository. The workflow also includes an initial step that accesses the contents of Supplementary Table 1, which are pulled from a Googles Docs spreadsheet ( https://docs.google.com/spreadsheets/d/11an55u9t2TAf0EV2pHN0vOd8Ww2Gie-tHp9xGULh_dA/edit?usp=sharing) to copy the specific data files to the system, and a step that extracts the volumes (in terms of number of voxels and absolute volume) of the resultant structures. The code for these additional steps is included in the GitHub repository as well. In this workflow, the following regions are assessed: brain and background (as determined from the masks generated by BET, the brain extraction tool), gray matter, white matter and CSF (from the output of FAST), and left and right accumbens, amygdala, caudate, hippocampus, pallidum, putamen, and thalamus-proper (from the output of FIRST) See Figure 2 for the workflow diagram.

***The execution environment.*** In order to utilize a computational environment that is, in principle, accessible to the other users in configuration identical to the one used to carry out this analysis, we created a Docker ( https://www.docker.com/) container to encapsulate the specific computation environment and analysis pipeline components: https://hub.docker.com/r/repronim/simple_workflow/tags/. A Docker container permits efficient environment and software delivery for easy deployment on most common operating systems (Linux, Windows, Mac). The build instructions for the Docker container are provided on the GitHub repository, and uses Debian 8.7 as the base operating system.

***Setting up the software environment on a different machine.*** In addition to a Docker container, one can re-execute the workflow on a different machine or cluster than the one used originally. General instructions for setting up the needed software environment on GNU/Linux and MacOS systems is provided in the README.md file in the GitHub repository. We assume FSL is installed and accessible on the command line (FSL can be found at https://fsl.fmrib.ox.ac.uk). In order to establish a precise overall environment, we use Conda ( https://conda.io/), a cross-platform package manager and handles user installations for many packages into a controlled environment. Unlike many operating system package managers (e.g., yum, apt), Conda does not require root privileges. This allows individuals to replicate isolated virtual environments easily without requiring system administrator help. Conda uses standard PATH variables to isolate the environments. Coupled with Anaconda cloud and conda-forge, Conda is capable of installing versioned dependencies of Python and other packages. In this way, a Python 2.7.12 environment can be set up and the Nipype workflow re-executed with a few shell commands, as noted in the README.md.

One can also use the NITRC Computational Environment (NITRC-CE, RRID:SCR_002171). The NITRC-CE is built upon NeuroDebian (RRID:SCR_004401; Hanke & Halchenko, 2011), and comes with FSL (version 5.0.9-3~nd14.04+1) pre-installed on an Ubuntu 14.04 operating system. We run the computational environment on the Amazon Web Services (AWS) elastic cloud computing (EC2) environment. With EC2, the user can select properties of their virtual machine (number of cores, memory, etc.) in order to scale the power of the system to their specific needs. For this paper, we used the NITRC-CE v0.42, with the following specific identifier (AMI ID): ami-ce11f2ae.

### The reference run

We performed the analysis (the above described workflow applied to the above described data, using the described computational system) with the Docker container provided and stored these results in our GitHub repository as the ‘reference run’, representing the official result that we are publishing for this analysis.

***Generating the reference run.*** In order to run the analysis, we executed the following steps:

1) Download the Docker image

> docker pull repronim/simple_workflow:1.1.0
2) Run the Docker image as follows to perform the analysis:

> docker run -it --rm –v \
   $PWD/output:/opt/repronim/simple_workflow/scripts/output \
   repronim/simple_workflow:1.1.0 run_demo_workflow.py \
      --key 11an55u9t2TAf0EV2pHN0vOd8Ww2Gie-tHp9xGULh_dA

***Exact re-execution.*** In principle, any user could run the analysis steps, as described above, to obtain an exact replication of the reference results. The similarity of this result and the reference result can be verified by running the following command:



                        > python check_output.py
                    


This program will compare the new results to the archived reference results and report on any differences, allowing for a numeric tolerance of 1e-6. If differences are found, a comma separated values (CSV) file is generated that quantifies these differences. The threshold in the ‘check_output.py’ script is simply selected to catch the presence of any numerical difference between the test run and the reference run. We discuss the implications of any differences, if found, below.

***Re-execution on other systems.*** While the reference analysis was run using the provided Docker container, this analysis workflow can be run, locally or remotely, on many different operating systems. In general, the exact results of this workflow depends on the exact operating system, hardware, and the software versions. Execution of the above commands can be accomplished on any other Mac OS X or GNU/Linux distribution, as long as FSL is installed. In these cases, the results of the ‘python check_output.py’ command may indicate some numeric differences in the resulting volumes. In order to demonstrate these potential differences, we ran this identical workflow on the Mac OS X 10.12.4, CentOS 7.3, and NITRC Computational Environment on AWS.

### Continuous integration

In addition to the reference run, the code for the project is housed in the Github repository. This allows integration with external services, such as CircleCI ( http://circleci.com), which can re-execute the computation every single time a change is accepted into the code repository. Currently, the continuous integration testing runs on amd64 Debian (8.7) and uses FSL (5.0.9) from NeuroDebian. This re-execution generates results that are compared with the reference run, allowing us to evaluate a similar analysis automatically.

## Results

### Exact versions of data, code, environment details, and output

The specific versions of data used in this publication are available from NITRC. The code, environment details, and reference output are all available from the GitHub repository. The results of the reference run are stored in the expected_output folder of the GitHub repository at https://github.com/ReproNim/simple_workflow/tree/1.1.0/expected_output. By sharing the results of this reference run, as well as the data workflow, and a program to compare results from different runs, we can enable others to verify that they can arrive at the exact same result (if they use the exact same execution environment), or how close they come to the reference results if they utilize a different computational system (that may differ in terms of operating system, software versions, etc.).

### Comparison of reference run and execution on other environments

When the workflow is re-executed in the same fashion (using the supplied Docker container) there is no observed difference in the output, regardless of Linux or Mac base platform. We also compared the execution of the reference run and re-execution natively (i.e. not via Docker container) in a separate MacOS environment. Table 1 indicates the numerical differences found when running natively on this MacOS example. Re-execution of the native analysis on the CentOS 7.3 and NITRC-CE (Ubuntu 14.04) on AWS provides an identical result to the reference run.

**Table 1.** Summary volumetric results from the simple workflow for the 24 subjects. Results are shown from the reference run (Docker) and a comparison run executed on a Mac OS X (10.12.4) system. The mean differences between these two systems are also summarized..

		Reference Run (Docker)		Mac OSX (10.12.4)			Difference				
Hemisphere	Region	Mean Volume (mm3)	STD	Mean Volume (mm3)	STD	Correlation	Mean Volume (mm3)	STD	Range	Mean Absolute Volume	Percent of Reference
**Left**	Accumbens	471.2	178.8	468.4	181.0	0.980	2.8	35.7	[-72.0, 75.6]	25.8	5.5
	Amygdala	840.2	257.9	849.2	262.9	0.963	-9.1	70.8	[-303.0, 84.0]	32.1	3.8
	Caudate	3842.4	650.4	3840.5	630.7	0.980	2.0	130.9	[-320.6, 518.4]	56.1	1.5
	Hippocampus	3276.2	794.9	3264.5	783.6	0.997	11.7	62.0	[-147.0, 185.0]	44.2	1.3
	Pallidum	1683.5	316.0	1668.8	313.6	0.995	14.7	30.7	[-16.8, 99.0]	20.2	1.2
	Putamen	4881.1	965.4	4890.2	952.7	0.998	-9.2	59.5	[-145.0, 144.0]	45.4	0.9
	Thalamus	8088.6	1240.8	8108.8	1239.0	0.998	-20.2	71.4	[-135.0, 194.0]	54.5	0.7
**Right**	Accumbens	389.1	147.6	402.2	148.5	0.966	-13.1	38.5	[-145.5, 32.0]	24.1	6.2
	Amygdala	882.3	297.8	897.6	290.7	0.918	-15.3	119.3	[-464.0, 221.0]	68.2	7.7
	Caudate	3781.0	762.1	3780.3	767.7	0.999	0.7	27.1	[-60.1, 48.0]	20.6	0.5
	Hippocampus	3433.4	796.9	3453.3	793.2	0.997	-19.9	60.6	[-190.0, 78.0]	42.4	1.2
	Pallidum	1694.5	293.8	1695.2	289.5	0.997	-0.7	21.8	[-64.0, 60.9]	12.9	0.8
	Putamen	4965.3	1017.3	4942.8	1008.0	0.993	22.5	118.0	[-201.6, 397.0]	78.4	1.6
	Thalamus.Proper	7723.6	1120.6	7724.3	1129.4	0.999	-0.7	52.9	[-145.0, 118.0]	33.6	0.4
**Total**	CSF	189856.5	26936.5	190209.3	26668.1	0.999	-352.8	1025.2	[-4274.2, 20.0]	359.7	0.2
	Gray Matter	684781.8	86048.8	684111.7	86173.4	1.000	670.1	2009.7	[-11.3, 7113.4]	676.3	0.1
	White Matter	513866.1	52348.3	513802.8	52341.8	1.000	63.3	467.3	[-541.2, 2192.4]	125.3	0.0
	Brain	1388504.4	132850.8	1388123.8	132962.5	1.000	380.6	1511.6	[-534.0, 6956.6]	425.1	0.0

## Discussion

Re-executability is an important first step in the establishment of a more comprehensive framework of reproducible computing. In order to properly compare the results of multiple papers, the underlying details of processing are essential to know to interpret the causes of ‘similarity’ and ‘dissimilarity’ between findings. By explicitly including linkage between a publication, and its data, workflow, execution environment and results, we can enhance the ability of the community to examine the issues related to reproducibility of specific findings.

In this publication, we are not looking at the causes of operating system dependence of neuroimaging results, but rather to emphasize the presence of this source of analysis variation, and examine ways to reduce this source of variance. Detailed results of neuroimaging analyses have been shown to be dependent on the exact details of the processing, specific computational operating system and software version ( Glatard *et al.*, 2015; Gronenschild *et al.*, 2012). In this work, we replicate the observation that, despite an exact match on the data and workflow, the results of analysis can differ between execution in different operating systems. The implications of these differences are complex. On the one hand, the correlation of the volumetric results within each individual subject, and in aggregate across the population is very high (0.918 - 1.000). On the other hand, we see, per structure, a range of volumetric differences that reveals a large span of percentage of structure differences, differences that are, not surprisingly, dependent upon the overall size of the structure itself. The extremes of this distribution of the average difference (provided in Table 1) can be as large as 7.7% for the right amygdala. Sources of volumetric variance in this range can be troubling as biological changes on this order of volumetric difference can otherwise be the types of changes that studies are designed to observe. While in this case, the volumetric differences are not numerically large, it illustrates the general nature of this overall concern.

Publications can be made re-executable relatively simply by including links to the data, workflow, and execution environment. A re-executable publication with shared results is thus ***verifiable,*** by both the authors and others, increasing the trust in the results. The current simple example shows a simple volumetric workflow on a small dataset in order to demonstrate the way in which this could work in the real world. We felt it important to document this on a small problem (in terms of data and analysis complexity) in order to encourage others to actually verify these results, which is a practice we would like to see become more routine and feasible in the future. While this example approach is ‘simple’ in the context of what it accomplishes, it is still a rather complex and *ad hoc* procedure to follow. As such, it provides a roadmap for improvement, simplification, and standardization of the ways that these descriptive procedures can be handled.

Progress in simplifying this simple example can be expected in the near future on many fronts. Software deployments that are coupled with specific execution environments (such as Docker, Vagrant, Singularity, or other virtual or container machine instances) are now being deployed for common neuroimaging applications. In addition, more standardized data representations (such as BIDS, Gorgolewski *et al.*, 2016; NIDM, Keator *et al.*, 2013; BDBags, http://bd2k.ini.usc.edu/tools/bdbag/) will simplify how experimental data is assembled for sharing and use in specific software applications. Data distributions with clear versioning of the data, such as DataLad ( http://datalad.org), will unify versioned access to data resources and sharing of derived results. While the workflow in this case is specified using Nipype, extensions to LONI Pipeline, shell scripting, and other workflow specifications is easily envisioned. Tools necessary to capture local execution environments (such as ReproZip, http://reprozip.org) will help users to share the software environment of their workflows in conjunction with their publications more easily.

## Conclusion

We have demonstrated a simple example of a fully re-executable publication to take publically available neuroimaging data and compute some volumetric results. This is accomplished by augmenting the publication with four ‘supplementary’ files that include exact specification of 1) data, 2) workflow, 3) execution environment, and 4) results. This provides a roadmap to enhance the reproducibility of neuroimaging publications, by providing a basis for verifying the re-executability of individual publications and providing a more structured platform to examine the generalizability of the findings across changes in data, workflow details and execution environments. We expect these types of publication considerations to advance to a point where it can be relatively simple and routine to provide such supplementary materials for neuroimaging publications.

## Software and data availability

Workflow and results are available on GitHub at: https://github.com/ReproNim/simple_workflow.

Archived workflow and results as at time of publication: doi, 10.5281/zenodo.800758 ( Ghosh *et al.*, 2017).

License: Apache License, Version 2.0.

The data used in this publication are available at NITRC-IR (project, fcon_1000; image collection: doi, 10.18116/C6C592 - Kennedy, 2017) and referenced in Supplementary File 1. These data were originally gathered from the NITRC-IR, 1000 Functional Connectomes project, Ann Arbor and New York sub-projects.

## Consent

The data used is anonymized and publicly available at NITRC-IR. Consent for the data sharing was obtained by each of the sharing institutions.

## Supplementary material

**Supplementary File 1: Data specification file.** This file contains the basic demographics of the subjects (Subject, Age, Hand, Gender, and Acquisition Site) as well as the URL to the imaging data, as hosted at NITRC-IR (project, fcon_1000).
