# Supplementary material for: A very simple, re-executable neuroimaging publication
Source: F1000Res. 2017 Jun 15;6:124. Originally published 2017 Feb 10. [Version 2] doi: 10.12688/f1000research.10783.2 (PMC5516225; doi:10.12688/f1000research.10783.2)
Supplement: Peer review history

# Peer review: Referee response for version 2

**DOI:** 10.5256/f1000research.12766.r24057

**Published:** 2017-07-24

**Authors:** Ze Wang

**Affiliations:** 1 Department of Radiology, Temple University, Philadelphia, PA, USA

The authors showed a framework to pack up the data, software, and analysis methods, specially for neuroimaging study. The results are reproducible which is the major aim of this paper. While I do think this paper is of interest to the general neuroimaging field, we should also notice that complete replication needs the data, software, and analysis procedures. The major hurdle however is often the data since data release may be sensitive and prohibited by regulations. How would the framework described in this paper be helpful for that case should be discussed.  Is it possible to have software securely access the data but not leak the data (meaning prohibiting downloading, transfering out of the database etc)?

I have read this submission. I believe that I have an appropriate level of expertise to confirm that it is of an acceptable scientific standard, however I have significant reservations, as outlined above.

# Peer review: Referee response for version 2

**DOI:** 10.5256/f1000research.12766.r24058

**Published:** 2017-07-17

**Authors:** Chao-Gan Yan

**Affiliations:** 1 CAS Key Laboratory of Behavioral Science, Institute of Psychology, Bejing, China

This is an exciting endeavor to address the reproducibility crisis by providing re-executable neuroimaging publication. This study is well designed, analyzed and written, with the analysis can be easily replicated. I only have some minor comments.

  

1. One may would like to follow this paper to try to make their publication re-executable. As the first step is sharing data, the authors may would like to provide some guidance to share data.
2. As the paper entitled “very simple”, the analysis here is structural image analysis without any statistical group comparison. I wholehearted look forward to the authors’ future progress on functional imaging. The flexibility of functional imaging is much more than structural imaging, thus the authors may would like to provide some discussion on that.
3. Group comparison and thresholding (with multiple comparison correction) are challenging to be reproduced, thus recently have been questioned a lot (including Eklund *et al.*, 2016 PNAS ^1^). The authors’ re-executable strategy may have some potential to prevent lots of p-hacking practices, thus deserve some discussion.

I have read this submission. I believe that I have an appropriate level of expertise to confirm that it is of an acceptable scientific standard.

## References

[1] EklundANicholsTEKnutssonH: Cluster failure: Why fMRI inferences for spatial extent have inflated false-positive rates. *Proc Natl Acad Sci U S A*.2016;113(28) : 10.1073/pnas.1602413113 7900-5 10.1073/pnas.1602413113 27357684PMC4948312

# Peer review: Referee response for version 2

**DOI:** 10.5256/f1000research.12766.r23503

**Published:** 2017-06-26

**Authors:** Konrad Hinsen

**Affiliations:** 1 Centre de Biophysique Moléculaire (UPR4301 CNRS), Rue Charles Sadron, Orléans, France; 2 Division Expériences, Synchrotron SOLEIL, Saint Aubin, France

The revision of the manuscript is based on an extensive rewrite of the underlying software environment. Platform independence has been enhanced significantly by the use of a Docker container for producing the reference results. The instructions for the software environment have been improved as well. Moreover, using the authors' Docker image does not require me to accept any license conditions. My main criticism of this work has been resolved: I can easily reproduce the authors' results, and I feel confident that others can do it as well.

 The manuscript itself has also improved significantly, and most issues raised by the two reviewers have been addressed satisfactorily.

 The only aspect which I still consider unsatisfactory is the discussion of numerical discrepancies. The manuscript still mentions a "numeric tolerance of 1e-6", without saying if this is an absolute or a relative tolerance. An inspection of the Python script check_output.py reveals that this tolerance value is not used explicitly there. The reference and actual values are compared using the NumPy function allclose(), which, according to its documentation, applies an absolute tolerance of 1.e-8 when called as in the script. The authors should (1) use an explicit threshold in their script, rather than relying on a default value that may change in future versions of NumPy and (2) quote this same value in the manuscript.

 Furthermore, the only explanation given for the choice of threshold is "The threshold in the ‘check_output.py’ script is simply selected to catch the presence of any numerical difference between the test run and the reference run." Catching *any* numerical difference would imply a threshold of zero. The purpose of a threshold is to distinguish between "small" differences that are most likely due to platform-dependent roundoff from "significant" differences whose cause needs to be investigated. The choice of threshold therefore depends both on the numerical algorithms (potential for roundoff discrepancies) and on the scientific interpretation of the results (how big must a change be to influence the conclusions?), which is what should be explained in the article.

I have read this submission. I believe that I have an appropriate level of expertise to confirm that it is of an acceptable scientific standard, however I have significant reservations, as outlined above.

# Peer review: Referee response for version 1

**DOI:** 10.5256/f1000research.11627.r20114

**Published:** 2017-03-06

**Authors:** Allan J. MacKenzie-Graham

**Affiliations:** 1 Division of Brain Mapping, Department of Neurology, University of California, Los Angeles, Los Angeles, CA, USA

I found the paper to be clear and straightforward, easy to read and understand.  I find the use of a NITRC-CE virtual machine as an execution environment in combination with Nipype an excellent mechanism for facilitating re-executability and documentation of a series of processing steps.  Combined with the use of GitHub to keep track of the elements, I believe that this is a remarkably good and fairly easily implemented solution.  This encapsulation is excellent for re-execution of an analysis or for applying exactly the same analysis to a novel set of data, however, as the authors state, it only begins to address reproducibility and generalizability across multiple execution environments.

 In this context, it is not clear to me what the processing environment was on the Mac OS comparison test, specifically what version of the OS and what version of the FSL tools were used in each case.  Second, Ubuntu 12.04 is used within NITRC-CE v0.42, but the Mac OS comparison appears to be done against a machine running Ubuntu 16.04.  I assume that 16.04 is a typo, but it should be corrected - and if it is not a typo, then a justification for a change in OS version should be given.  In previous work by myself ^1^ and others ^2^, we showed that differences in software version and compilation settings can lead to measurably different results.  A statement expressing the software versions used and that the software was compiled using similar settings (e.g. same level of optimization, same architecture was used - x86 or x64, etc.) would be reassuring.  I realize that this similarity in environment is implied by the mechanism used to install the software, but it should be stated explicitly.

 Overall, this is an excellent manuscript and implementation for sharing re-executable neuroimaging results.  The methods and reporting can readily be repeated and replicated, supporting the main thrust of the paper.

I have read this submission. I believe that I have an appropriate level of expertise to confirm that it is of an acceptable scientific standard, however I have significant reservations, as outlined above.

## References

[1] Mackenzie-GrahamAJVan HornJDWoodsRPCrawfordKLTogaAW: Provenance in neuroimaging. *Neuroimage*.2008;42(1) : 10.1016/j.neuroimage.2008.04.186 178-95 10.1016/j.neuroimage.2008.04.186 18519166PMC2664747

[2] GronenschildEHHabetsPJacobsHIMengelersRRozendaalNvan OsJMarcelisM: The effects of FreeSurfer version, workstation type, and Macintosh operating system version on anatomical volume and cortical thickness measurements. *PLoS One*.2012;7(6) : 10.1371/journal.pone.0038234 e38234 10.1371/journal.pone.0038234 22675527PMC3365894

# Response

**Published:** 2017-05-31

**Authors:** David Kennedy

**Affiliations:** University of Massachusetts Medical School, USA

**We thank the reviewer for their thoughtful review and helpful comments. We have revised the manuscript and design of this manuscript to meet many of the concerns raised, and we believe that this has resulted in an improved presentation. We have also reworked the repository and the way the experiment can be reproduced and extended.**

 Reviewer Comment: In this context, it is not clear to me what the processing environment was on the Mac OS comparison test, specifically what version of the OS and what version of the FSL tools were used in each case.  

**Response: In the various ‘comparison runs’ that we present, we now take more care in providing complete descriptions of the OS and software versions that are being used.**

 Reviewer Comment: Second, Ubuntu 12.04 is used within NITRC-CE v0.42, but the Mac OS comparison appears to be done against a machine running Ubuntu 16.04.  I assume that 16.04 is a typo, but it should be corrected - and if it is not a typo, then a justification for a change in OS version should be given.  In previous work by myself 1 and others 2, we showed that differences in software version and compilation settings can lead to measurably different results.  A statement expressing the software versions used and that the software was compiled using similar settings (e.g. same level of optimization, same architecture was used - x86 or x64, etc.) would be reassuring.  I realize that this similarity in environment is implied by the mechanism used to install the software, but it should be stated explicitly.

**Response: As above, there is clearly some ambiguity as to how we presented the ‘comparison runs’ in the original manuscript that we try to clarify in this version. In the original, the NITRC-CE AWS Ubuntu 12.04 FSL 5.0.9 run was the ‘reference’ and we compared to runs of this workflow (using FSL 5.0.9 in all cases) on local MAC OS 10.10.4, and Ubuntu 16.04  platforms. In the current version, we add a Docker version of the of the workflow (FSL 5.0.9, Debian jessie (8.7)), and enhance the description of the comparison runs. Finally, thanks for the additional references which we now also include.**

# Peer review: Referee response for version 1

**DOI:** 10.5256/f1000research.11627.r20292

**Published:** 2017-02-21

**Authors:** Konrad Hinsen

**Affiliations:** 1 Centre de Biophysique Moléculaire (UPR4301 CNRS), Rue Charles Sadron, Orléans, France; 2 Division Expériences, Synchrotron SOLEIL, Saint Aubin, France

This article aims to demonstrate how a neuroimaging study can be published in such a way that readers can re-execute the complete workflow with (relative) ease. Although the concrete study used as an example is probably of little scientific interest, it could serve as a template and guideline for other researchers using the same software tools.

 My main issue with this paper is that I was unable to re-execute the workflow, in spite of the authors' efforts to document the process. A detailed technical explanation is provided at the end of this review. In addition to technical obstacles that could in principle be overcome, the main issue is the use of the proprietary software package FSL, whose licence can be interpreted as prohibiting its use in the context of a review for F1000Research. It is not clear to me if using FSL via NeuroDebian would somehow solve this problem, because I did not succeed in obtaining the NeuroDebian docker image.

 Another issue of the interpretation of numerical tolerances. The article says that the authors made comparisons "allowing for a numeric tolerance of 1e-6", but no explanation is given for this particular choice, nor is it said if this is an absolute or a relative error criterion. Table 1 shows numerical results obtained on different platforms, but provides no guide for their interpretation. How big would a difference have to to influence the scientific interpretation of the results?

 Finally, readers wishing to take this example as a starting point for preparing their own studies reproducibly would benefit from a more extensive discussion of the technical choices and of the work required for actually composing, rather than simply consulting, the authors' code repository. For example, it is not stated explicitly anywhere that the workflow takes the form of a Python script (run_demo_workflow.py). The use of conda in re-creating an environment with precise versions of each software package is also not generally known and deserves more explanation.

**Attempting to re-execute the example workflow**

 I tried to re-execute the workflow on a MacBookPro running under macOS 10.11.6, using both procedures explained in the authors' README.md.

*1. "Within your current environment".*

 The first obstacle is "Make sure FSL is available in your environment and accessible from the command line." Where do I get FSL? Which version(s) are acceptable? Please provide at the very least a link to the software's Web page (https://fsl.fmrib.ox.ac.uk/).

 I decided not to install FSL on my computer because I am not willing to accept the licence. It excludes commercial use, and in particular "use of the Software to provide any service to an external organisation for which payment is received". F1000Research offers reviewers a reduction on future article page charges, which could be interpreted as a form of payment. Beyond this specific legal issue, I also consider it unreasonable to request reviewers to register as users of proprietary software, providing personal data for marketing purposes in the process.

 I did, however, continue the setup process to check it for completeness. The instruction "If you already have a `conda` environment, please follow the detailed steps below." lacks some precision: where exactly do I have to start if I already have a conda environment? The right answer is "at 'conda config --add channels conda-forge'", which I think is not obvious.

*2. "Within Docker"*

 Running the Simple_Prep_docker under macOS ends with the error message

 readlink: illegal option -- f

 usage: readlink [-n] [file ...]

 I modified the script, replacing "readlink" by "greadlink" from Homebrew's coreutils package. The next error message then is

 sed: -i may not be used with stdin

 There are two uses of "sed -i" in the script, but for neither one it is obvious under which conditions it would erroneously act on stdin. I decided to give up at this point. Considering the use of apt-get in the script, it probably requires Debian or Ubuntu Linux anyway.

 I am not sure the authors can do much to address this issue, given that writing platform-independent shell scripts is difficult to impossible, but they should at least say clearly in the installation instructions for which platforms they have actually tested the installation.

I have read this submission. I believe that I have an appropriate level of expertise to confirm that it is of an acceptable scientific standard, however I have significant reservations, as outlined above.

# Response

**Published:** 2017-05-31

**Authors:** David Kennedy

**Affiliations:** University of Massachusetts Medical School, USA

**We thank the reviewer for their thoughtful review and helpful comments. We have revised the manuscript and design of this manuscript to meet many of the concerns raised, and we believe that this has resulted in an improved presentation. We have also reworked the repository and the way the experiment can be reproduced and extended.**

 Reviewer Comment: My main issue with this paper is that I was unable to re-execute the workflow, in spite of the authors' efforts to document the process. A detailed technical explanation is provided at the end of this review.

**Response: We are sorry that this did not work as expected in your case. As further detailed in our response to your detailed technical explaination below, and in the github ‘issue’ ( https://github.com/ReproNim/simple_workflow/issues/15), we hope that we have successfully ammended the procedure to make the system even more re-executable by including the Docker re-execution option.**

 Reviewer Comment:  In addition to technical obstacles that could in principle be overcome, the main issue is the use of the proprietary software package FSL, whose licence can be interpreted as prohibiting its use in the context of a review for F1000Research. It is not clear to me if using FSL via NeuroDebian would somehow solve this problem, because I did not succeed in obtaining the NeuroDebian docker image.

**Response: We agree that licensing issues are very important to consider. Many of the users in the neuroimaging community have FSL locally, and have consented to the FSL licensing terms; hence re-execution of this workflow incurs no additional licensing issues. Users using a NITRC-CE AWS instance are explicitly presented a page that that details the licensing terms of the software installed on that instance and use of the instance is with the acknowledgement of the licensing terms. Our initial Docker did not present these licensing terms, but in our new README for the repository and Docker, we have include a notice for commercial use.**

****

 Reviewer Comment: Another issue of the interpretation of numerical tolerances. The article says that the authors made comparisons "allowing for a numeric tolerance of 1e-6", but no explanation is given for this particular choice, nor is it said if this is an absolute or a relative error criterion. Table 1 shows numerical results obtained on different platforms, but provides no guide for their interpretation. How big would a difference have to to influence the scientific interpretation of the results?

**Response: We now provide more information on this issue. The threshold we apply in the ‘check_output.py’ script is simply selected to catch the presence of any numerical difference between the test run and the reference run.  The default in the version of numpy used is in the present container is 1e-5. The biological interpretation of these differences is multi-faceted. On the one hand, the correlation of the volumetric results within each individual subject, and in aggregate across the population is very high (0.918 - 1.000). On the other hand, we see, per structure, a range of volumetric differences that reveals a large span of percentage of structure differences, differences that are, not surprisingly, dependent upon the overall size of the structure itself. The extremes of this distribution of the average difference (provided in Table 1) can be as large as 7.7% for the right amygdala. Sources of volumetric variance in this range can be troubling as biological changes on this order of volumetric difference can otherwise be the types of changes that studies are designed to observe.**

****

 Reviewer Comment: Finally, readers wishing to take this example as a starting point for preparing their own studies reproducibly would benefit from a more extensive discussion of the technical choices and of the work required for actually composing, rather than simply consulting, the authors' code repository. For example, it is not stated explicitly anywhere that the workflow takes the form of a Python script (run_demo_workflow.py). The use of conda in re-creating an environment with precise versions of each software package is also not generally known and deserves more explanation.

**Response: We now add more discussion of the topic of approaches that others can take to generate more re-executable workflows, and the various challenges in representing the execution environment.  Specifically, conda (https://conda.io/) is a cross-platform package manager and handles user installations for many packages into a controlled environment. Unlike many operating system package managers (e.g., yum, apt), Conda does not require root privileges. This allows individuals to replicate isolated virtual environments easily without requiring system administrator help. Conda uses standard PATH variables to isolate the environments. Coupled with Anaconda cloud and conda-forge, Conda is capable of installing versioned dependencies of Python and other packages.**

 Reviewer Comment: Reviewer Attempting to re-execute the example workflow

 The first obstacle is "Make sure FSL is available in your environment and accessible from the command line." Where do I get FSL? Which version(s) are acceptable? Please provide at the very least a link to the software's Web page ( https://fsl.fmrib.ox.ac.uk/).

**Response: Links are now provided, and we remind the reader of this page that 5.0.9 is the version of the reference run. Again, half of the point of this exercise is to provide the opportunity to see what happens, numerically, IF the user is already using a different environment or version.**

 Reviewer Comment: I decided not to install FSL on my computer because I am not willing to accept the licence. It excludes commercial use, and in particular "use of the Software to provide any service to an external organisation for which payment is received". F1000Research offers reviewers a reduction on future article page charges, which could be interpreted as a form of payment. Beyond this specific legal issue, I also consider it unreasonable to request reviewers to register as users of proprietary software, providing personal data for marketing purposes in the process.

**Response: For these various reasons, we have now elected to also include a Docker version of this workflow that precludes the need to locally download specific software to one’s local computer. We have also included a commercial use statement from the FSL developers in the README. While most members of the neuroimaging community have access to FSL, in the future, we hope to move to FOSS versions of imaging tools to make such testing accessible to a broader community.**

****

 Reviewer Comment:​​​​​​​ I did, however, continue the setup process to check it for completeness. The instruction "If you already have a `conda` environment, please follow the detailed steps below." lacks some precision: where exactly do I have to start if I already have a conda environment? The right answer is "at 'conda config --add channels conda-forge'", which I think is not obvious.

**Response: We have updated the script to test for existence of software and create a standalone environment that does not interfere with any user environment directly. The user can activate this environment if the user so chooses. Since FSL download on non-Debian systems requires going to a site, we simply recommend that people do so on their own. The same script is also used in the Docker container. We have updated this in the README for the repository.**

 Reviewer Comment: 2. "Within Docker"

 etc.

 I am not sure the authors can do much to address this issue, given that writing platform-independent shell scripts is difficult to impossible, but they should at least say clearly in the installation instructions for which platforms they have actually tested the installation.

**Response: We thank the reviewer for alerting us to this compatibility issue.  As we have discussed in the github issue, as a workaround, we first  have pre-generated a Dockerfile so that reviewer could run the analysis and validate our findings.  We have recently finalized our changes to the script to make it compatible with both Linux and OSX so it should now run natively on reviewers infrastructure without issues.  We very much appreciate the time and effort the reviewer took to help finalize this script.**
